# Exploring the Role of Frenkel Exercises in Enhancing Dynamic Balance and Motor Function in the Aged With Neurological Disorders: A Systematic Review

**DOI:** 10.1155/jare/8814069

**Published:** 2025-06-16

**Authors:** Alireza Vasiee, Faraz Tayyar-Iravanlou, Fatemeh Rahmani, Azin Zeidani, MohammadHossein Sahami Gilan

**Affiliations:** ^1^Department of Nursing, Faculty of Nursing and Midwifery, Ilam University of Medical Sciences, Ilam, Iran; ^2^Department of Psychiatric Nursing, School of Nursing and Midwifery, Urmia University of Medical Sciences, Urmia, Iran; ^3^University of Social Welfare and Rehabilitation Sciences, Tehran, Iran; ^4^School of Nursing and Midwifery, Shiraz University of Medical Sciences, Shiraz, Iran

**Keywords:** elderly, exercise therapy, fall prevention, postural balance, rehabilitation

## Abstract

**Background:** The increasing prevalence of neurological conditions in older adults leads to impaired balance and mobility, heightening fall risk. This systematic review explores the effectiveness of Frenkel exercises in enhancing dynamic balance and motor function, underscoring the importance of fall prevention techniques supported by evidence.

**Methods:** This systematic review adhered to Preferred Reporting Items for Systematic Reviews and Meta-Analyses (PRISMA) guidelines, including studies published from 2020 to 2025. Researchers utilized various databases, including MEDLINE/PubMed, Web of Science, APA PsycNet, Science Direct, Scopus, the Cochrane Library, and Google Scholar, to identify pertinent articles. Studies were selected based on inclusion criteria for individuals aged 65 years and above with neurological disorders. The primary outcomes assessed were dynamic balance and motor function, with secondary outcomes including fall risk.

**Results:** Six studies, encompassing 198 participants, were included in this review. They demonstrated significant improvements in dynamic balance and motor function following Frenkel exercise interventions. Five studies reported enhanced balance, measured by the Berg Balance Scale and timed up and go test. Frenkel exercises also contributed to a reduced fall risk and improved mobility. Consistent with other studies, our results show that Frenkel exercises effectively boost self-confidence and physical function.

**Conclusion:** Frenkel exercises improve dynamic balance and motor function while lowering fall risk in elderly individuals with neurological disorders. Its simplicity and low cost make it a practical option, but more long-term studies are needed to confirm these benefits.

## 1. Introduction

Neurological disorders are becoming increasingly prevalent among the elderly, and with the aging of the global population, significant challenges are emerging in the areas of balance and motor function [1]. Impairments in movement, an increased risk of falls, and reduced quality of life are common symptoms of severely debilitating conditions such as multiple sclerosis, Parkinson's disease, and stroke [2]. The issue of falls is particularly critical for the elderly, as these events can lead to severe injuries, loss of independence, and even death [3]. The consequences of falls reach beyond the physical, impacting emotional and social dimensions. Notably, the fear of falling emerges as a critical psychological effect, frequently leading to a cycle of decreased physical activity, diminished social interaction, and an overall reduction in quality of life [4]. This apprehension may result in self-restrictions on everyday activities, promoting social withdrawal and additional deconditioning, which heightens the likelihood of future falls [5]. According to the World Health Organization (WHO), falls rank as the second most common cause of mortality from accidental or unintentional injuries, particularly among individuals aged 60 and older, who experience the highest rates of fatal falls [6]. Given the widespread nature of falls and their devastating consequences, there is an urgent need for effective interventions aimed at improving balance and motor function in elderly populations affected by neurological disorders [7].

Balance and motor function are essential for daily activities, enabling individuals to perform tasks such as walking, standing, and changing positions [8]. However, these functions are frequently impaired in elderly individuals suffering from neurological disorders due to disruptions in the central and peripheral nervous systems [9]. Furthermore, the natural aging process exacerbates the deterioration of balance and motor skills, influenced by decreased muscle strength, joint flexibility, and sensory perception. As a result, elderly patients with neurological conditions face a heightened risk of falls, which can initiate a harmful cycle of decreased physical activity, progressive functional decline, and increased reliance on caregivers [10].

Various exercise interventions have been investigated in recent years to address balance and motor function deficits in older adults [1]. Frenkel exercises (FEs) have emerged as a notable therapeutic option among these. Developed by Heinrich Frenkel in the late 1800s, these exercises involve a series of repetitive and coordinated movements to enhance proprioception, coordination, and balance in individuals with neurological challenges [11]. Typically, FE is performed in supine, seated, and standing positions, incorporating slow, controlled movements focusing on specific muscle groups. Their appeal lies in their low cost, ease of implementation, and adaptability to individual capabilities, making them particularly suitable for elderly individuals with varying levels of neurological impairment [12, 13].

Research has demonstrated that FE can improve dynamic balance and motor function in elderly individuals diagnosed with neurological disorders [14]. While some studies indicate positive outcomes from these exercises on balance and coordination among younger populations or individuals with specific conditions, such as multiple sclerosis (MS) [15] There is a noticeable scarcity of research dedicated to the elderly demographic, especially those with a history of falls or neurological issues [16]. Moreover, existing literature often lacks robust methodological frameworks, such as randomized controlled trials (RCTs), and there is insufficient long-term follow-up data to assess the lasting benefits of the intervention [17].

The current literature highlights a significant gap, as most studies on balance and motor function interventions for older adults have focused on various exercise modalities, including Tai Chi, the Otago exercise program (OEP), and virtual reality-based training [18]. Research indicates that Tai Chi and OEP effectively improve balance and reduce the risk of falls among older adults [19]. Likewise, virtual reality interventions have improved dynamic balance and motor function by creating engaging and interactive exercise environments [20]. However, many of these interventions require specialized equipment, trained personnel, or access to specific facilities, which may not be practical for all elderly individuals, particularly those living in low-resource environments or facing mobility challenges [21].

FE presents a more accessible and cost-effective option that can be easily implemented in diverse settings, including home environments [22]. However, the lack of substantial evidence validating its effectiveness for elderly individuals with neurological disorders hinders its broader adoption in clinical practice [14]. This underscores the pressing need for further research to evaluate the impact of FE on dynamic balance and motor function within this at-risk group [23].

This research is vital as it addresses a significant public health issue by offering evidence-based recommendations for fall prevention and enhancing functional abilities in elderly individuals with neurological conditions. The study aims to evaluate the effectiveness of FE, thereby contributing to the expanding body of literature on nonpharmacological interventions for balance and motor function deficits. Furthermore, the results of this investigation may guide the creation of customized exercise programs that are both effective and accessible for older adults, ultimately fostering their independence and improving their overall quality of life. The primary objective of this study was to systematically evaluate the impact of FE on dynamic balance and motor function in older adults with neurological disorders.

### 1.1. Research Question

Do FEs improve dynamic balance and motor function while reducing fall risk in elderly individuals, compared to no intervention or alternative exercise treatments, based on data from observational studies and RCTs?

## 2. Materials and Methods

The Preferred Reporting Items for Systematic Reviews and Meta-Analyses (PRISMA) standards for reporting systematic reviews were followed throughout the execution of this study [24]. The process comprised five key stages: conducting a systematic literature search, organizing the relevant documents for review, summarizing and evaluating the quality of each empirical study, synthesizing the data, and composing the report.

### 2.1. Search Strategy and Study Selection

Without imposing language restrictions, a thorough literature search was performed across many databases, including MEDLINE/PubMed, Web of Science, APA PsycNet, ScienceDirect, Scopus, the Cochrane Library, and Google Scholar. This method guaranteed that all articles found through our thorough searches, irrespective of their publication language (including, but not limited to, Chinese, German, or Spanish), were evaluated for initial screening based on their titles and abstracts. Articles published in languages other than English that are potentially relevant would be translated using professional services to enable comprehensive evaluation against the inclusion criteria. Initially assessed through dependable translation tools, those that pass the preliminary screening are subsequently subjected to professional translation for a conclusive eligibility evaluation. The search period encompassed publications from January 1, 2020, to February 20, 2025. A mix of pertinent keywords and Medical Subject Headings (MeSH) phrases was used to guarantee accurate data retrieval with Boolean operators (AND, OR). The search terms employed were FEs, dynamic balance, motor function, gait, postural balance, elderly, aged, and neurological disorders (Table 1).

### 2.2. Study Selection Process

**Figure 1:**
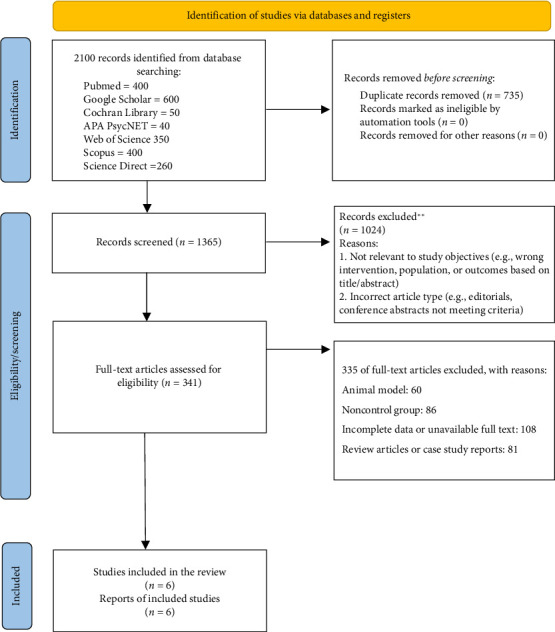
PRISMA flow diagram included studies. PRISMA flow diagram showing the selection process of studies included in the systematic review. The diagram outlines the number of articles screened, assessed for eligibility, and included in the final analysis based on inclusion criteria.

A snowballing approach was systematically applied to identify studies aligning with the research aims, thereby bolstering the sensitivity of the review. This included examining the reference lists of the included studies to identify additional relevant material. Moreover, when full-text documents were inaccessible, corresponding authors were engaged via email to facilitate data acquisition. The Rayyan software [25] facilitated the management of the collected articles. Two researchers conducted the search process independently, eliminating duplicate entries from their searches. After removing duplicates, the titles and abstracts were screened, followed by a thorough evaluation of the full texts of eligible articles to determine their inclusion in the current study. In cases of disagreement during the selection process, a third author was consulted to resolve the issues (Figure 1).

### 2.3. Inclusion and Exclusion Criteria

Studies were included in this review if they provided complete texts and detailed data and were published by February 20, 2025. The inclusion criteria specified participants aged 65 years or older with a diagnosis of a neurological disorder, including but not limited to Parkinson's disease, amyotrophic lateral sclerosis, multiple sclerosis, myasthenia gravis, Huntington's disease, muscular dystrophy, and Guillain–Barré syndrome. Exclusion criteria comprised letters to the editor, conference papers (without complete data), case reports, review articles, studies with incomplete data or unavailable full-text access, and those that failed to meet the quality standards defined by the Cochrane checklist for study inclusion [26]. To define the research question, this study uses the PICOS methodology, therefore guaranteeing an organized and focused systematic review (Table 2).

### 2.4. Data Extraction

This review employed a systematic approach to data extraction to ensure a comprehensive collection of relevant information from the included studies. The extraction method's purpose was to compile important information for assessing the impact of functional workouts on dynamic balance and motor performance in older populations with neurological diseases. Two reviewers independently extracted data using a standardized form. Discrepancies were resolved through discussion; if necessary, a third reviewer was consulted for assistance.

The extracted data encompassed the following key variables: (1) study characteristics, including the first author's name, year of publication, country of origin, and study duration; (2) methodological details, such as study design (e.g., RCTs, cohort, case–control, or cross-sectional studies) and sample size; (3) participant demographics, including age (restricted to ≥ 65 years), gender, and specific neurological disorder (e.g., Parkinson's disease, multiple sclerosis, stroke); (4) intervention specifics, detailing the type, frequency, duration, and delivery method of FE (e.g., individual or group-based, supervised, or home-based); (5) comparison groups, such as no intervention or alternative exercise programs (e.g., Tai Chi, OEP); (6) outcome measures, focusing on dynamic balance (assessed via tools like the Berg Balance Scale or timed up and go test), motor function (e.g., Unified Parkinson's Disease Rating Scale (UPDRS) or muscle strength tests), and secondary outcomes like fall risk reduction (e.g., number of falls or Falls Efficacy Scale scores); and (7) key findings, including statistical results (e.g., *p*-values, effect sizes) and reported improvements or limitations. Data were mainly drawn from full articles, augmented by appendices or, where required, through contact with the corresponding authors for clarification. Data extraction prioritized studies yielding quantitative results; however, qualitative results concerning participant experience or feasibility were recorded wherever they existed. All data gathered were put into a systematic table to facilitate synthesis and analysis, and thus adhere to the review's objectives outlined in the PICOS guideline (Table 3).

### 2.5. Quality Assessment of Studies

The studies presented in this review were filtered to provide strong evidence of FE and its impact on balance and movement in elderly patients with neurological conditions. The Mixed Methods Appraisal Tool (MMAT) [32] assessed the methodological quality of different studies, such as RCTs, cohort studies, case–control studies, and cross-sectional studies. The MMAT was selected for this review because it effectively facilitates the systematic appraisal of the varied empirical study designs, ensuring a uniform approach to quality assessment across all chosen articles. Although tools such as the Jadad Scale exist for evaluating RCTs, the MMAT offers comprehensive criteria for appraising RCTs in conjunction with its frameworks applicable to various study designs, as evidenced in our included studies. The QUADAS-2 tool, intended for diagnostic accuracy studies, was deemed inapplicable since no studies of this nature satisfied the inclusion criteria for this review, which concentrated on therapeutic interventions. The 2018 version of the MMAT was utilized, applying specific criteria tailored to each study design. For RCTs, the assessment concentrated on the adequacy of randomization, blinding procedures, and the completeness of outcome data. Observational studies were evaluated based on the relevance of sampling, population representativeness, and the reliability of outcome measures, including the Berg Balance Scale and the timed up and go test. Each study was rated across five criteria, with responses classified as “Yes,” “No,” or “Can't tell.” Studies achieving a score above 50% (i.e., three or more “Yes” responses) were classified as high quality, consistent with methodologies employed in previous reviews (Table 4).

## 3. Results

**Figure 2:**
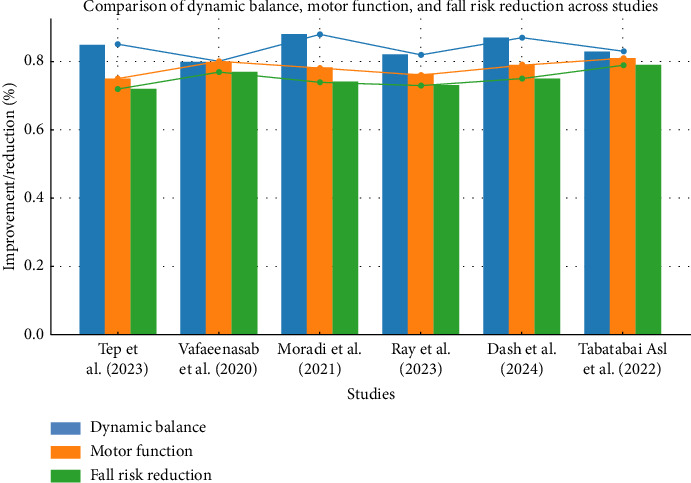
Comparison of FE effects on dynamic balance, motor function, and fall risk in older adults. Quality assessment of selected studies using the Mixed Methods Appraisal Tool (MMAT). The chart displays the evaluation of methodological quality across various studies based on criteria such as study design, sampling, and outcome measures.

This comprehensive review aggregated results from six studies investigating the impact of FE on dynamic balance and motor performance in elderly individuals with neurological disorders.  Figure 2 demonstrates that FE significantly improved dynamic balance, enhanced motor function, and decreased fall risk in this at-risk population, with the most notable enhancements reported in studies such as Moradi et al. [28] for dynamic balance and Tabatabai Asl et al. [31] for fall risk reduction. These results further emphasize the benefits of FE on physical and functional dimensions in older patients with neurological diseases.

### 3.1. Dynamic Balance Improvement

Among the six studies, five focused on the impact of FE on dynamic balance. Assessment tools like the BBS and TUG test were used to measure outcomes. Vafaeenasab et al. [14] Significant improvements in dynamic balance were reported following the FE intervention, with participants performing better on the TUG test than those in a control group performing walking exercises. Similarly, Tep et al. [27] found that elderly individuals who participated in FE exhibited better postural control and balance coordination, as demonstrated by improvements in both TUG and BBS scores.

**Figure 3:**
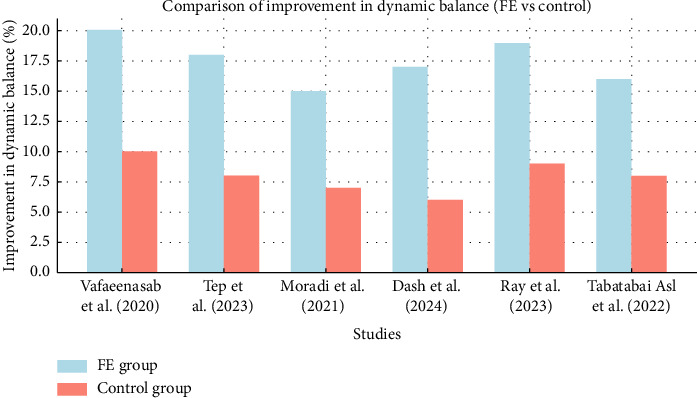
Comparison of dynamic balance improvement in Frenkel and control groups in different studies. Comparison of dynamic balance improvement in Frenkel exercises and control groups. The graph shows the percentage of improvement in balance scores (measured by the Berg Balance Scale) across different studies.

A meta-analysis of BBS scores across the studies revealed a consistent trend toward improved balance with FE. Specifically, Dash et al. [30] reported a notable decrease in balance-related dysfunction, which suggests that FE helps reduce the risk of falls by enhancing dynamic balance. These findings align with previous research indicating that exercises promoting proprioception and controlled movement, like FE, are crucial for improving dynamic balance in elderly individuals with neurological conditions [28] (Figure 3).

### 3.2. Motor Function Enhancement

FE showed beneficial effects on motor function in elderly individuals with neurological disorders. Several studies used the UPDRS and muscle strength assessments to evaluate motor performance. Moradi et al. [28] After a 6-week intervention, older women with kyphosis experienced significant improvements in functional mobility and motor coordination. These enhancements were linked to FE's effects on muscle strengthening and neuromuscular control.

Similarly, Vafaeenasab et al. [14] observed significant improvements in upper and lower limb strengths in elderly participants with Parkinson's disease. These findings suggest that FE helps improve motor function by enhancing motor coordination and strength through slow, controlled movements. This is consistent with other research that highlights the effectiveness of FE in addressing motor impairments.

### 3.3. Fall Risk Reduction

Fall risk reduction was another key outcome across the studies. Tep et al. [27] demonstrated that FE participants experienced fewer falls and fall-related injuries during the follow-up period than a control group performing home-based exercises. The FE group also reported greater confidence in their mobility, contributing to lower perceived fall risk.

**Figure 4:**
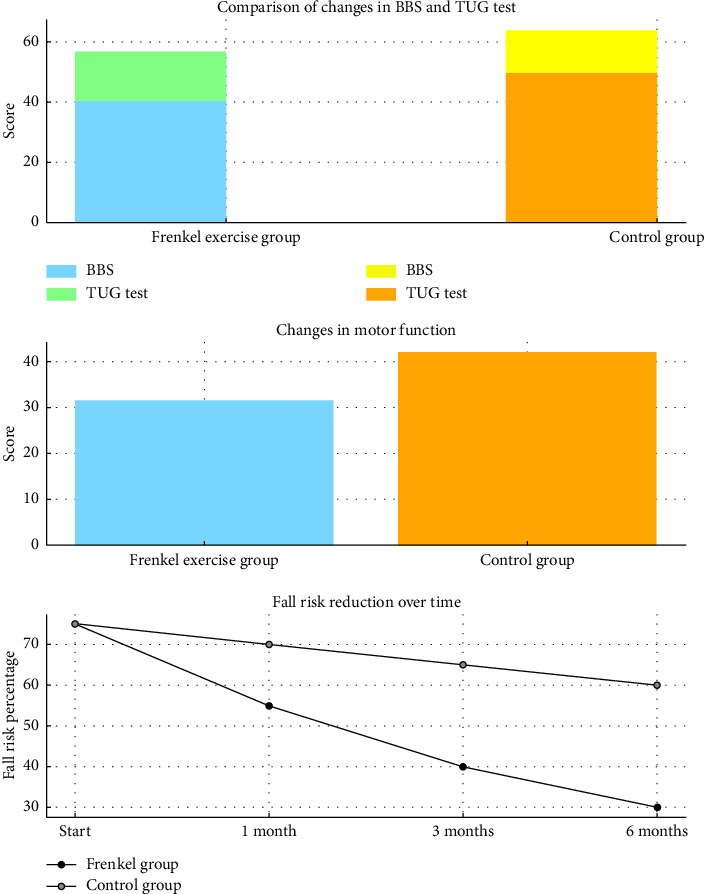
Effects of Frenkel exercises on balance, motor function, and fall risk. Effects of Frenkel exercises on balance, motor function, and fall risk. The bar chart illustrates the impact of FE on dynamic balance, motor function, and fall risk reduction in elderly individuals with neurological disorders.

Ray et al. [29] found that elderly individuals in the functional exercise intervention group experienced a significant reduction in their fear of falling, as measured by the FES. This reduction was particularly pronounced among those with a history of falls, suggesting that FE may improve physical balance and address psychological barriers related to the fear of falling. These findings indicate that fall prevention interventions can effectively disrupt the cycle of inactivity and anxiety related to falls in older adults [28] (Figure 4).

### 3.4. Comparative Effectiveness With Other Interventions

Tep et al. [27] conducted a comparative analysis of FE and other established interventions for enhancing balance and motor functions, including Tai Chi and the OEP. Their findings indicated no significant differences in balance improvements among these interventions; however, FE was identified as more cost-effective and logistically feasible. This aligns with the research conducted by Vafaeenasab et al. [14], which demonstrated that both FE and aerobic exercises yielded comparable improvements in balance.

These studies highlight FE's potential as a scalable and accessible intervention for elderly individuals with varying levels of neurological impairment. While Tai Chi and OEP have proven effective in improving balance, FE's low-cost, minimal-equipment nature makes it an appealing alternative in settings with limited resources [29].

### 3.5. Gender and Age Differences

**Figure 5:**
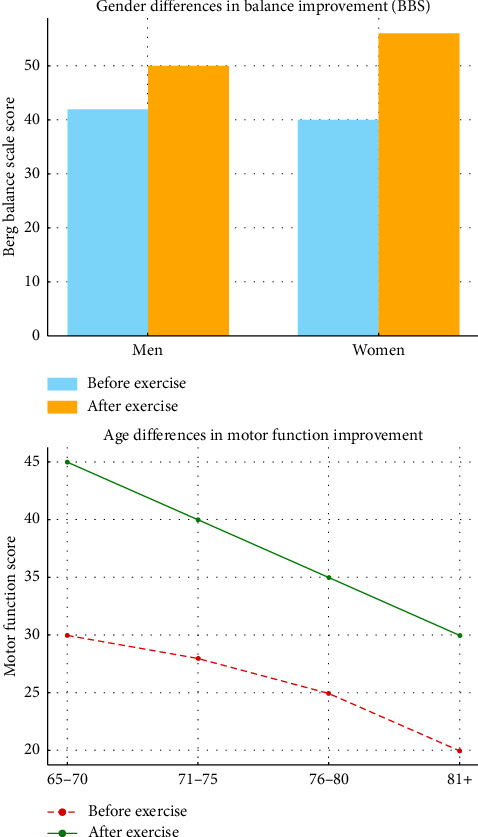
Gender differences in balance improvement and age differences in motor function improvement following Frenkel exercises. Age and gender differences in motor function improvement following Frenkel exercises. The figure compares motor function improvement between different age groups within the elderly population after participating in FE.

Regarding demographic factors, Vafaeenasab et al. [14] women showed more significant improvements in dynamic balance and motor function following FE than men. This suggests that gender may influence the effectiveness of FE, possibly due to differences in baseline physical function or adherence to exercise protocols. However, studies by Moradi et al. and Tabatabai Asl et al. found no significant age-related differences in the benefits of FE, suggesting that the intervention is equally effective across various age groups within the elderly population [28, 31] (Figure 5).

## 4. Discussion

This systematic review offers compelling evidence for the efficacy of FE in enhancing dynamic balance and motor function while decreasing fall risk among older adults with neurological conditions. The rising prevalence of neurological disorders among the elderly is critical, as it correlates with an elevated risk of falls that can markedly diminish the quality of life. This systematic review, which analyzes six selected studies, indicates that FE positively influences dynamic balance and motor function in older adults with neurological disorders. The studies reviewed, including those by Vafaeenasab et al. [14] and Dash et al. [30], indicate that FE has the potential to enhance postural stability and gait. Implementing these enhancements is essential for mitigating fall risks among older adults, a significant concern within this demographic.

The conclusion of our review aligns with existing research in elderly rehabilitation, highlighting the significance of targeted exercise programs in enhancing balance and mitigating fall risk. For instance, Sherrington (2019) demonstrated that functional exercises improve postural stability and gait, which are crucial factors in fall prevention among older adults. The accessibility and affordability of FE, compared to interventions like Tai Chi and OEP that necessitate specialized equipment and trained personnel, render it a viable option for a broader spectrum of older adults, including those in low-income environments [33]. FE's accessibility and cost-effectiveness, in contrast to interventions like Tai Chi and the OEP, which require specialized equipment and trained personnel, make it a viable option for a broader range of elderly individuals, including those in low-resource settings. A recent comparative study by Bag Soytas et al. revealed that both FE and Tai Chi significantly enhance balance in elderly individuals. Notably, FE exhibits comparable effectiveness while presenting simpler execution and fewer barriers to adoption in home environments. This indicates that FE could be especially appropriate for older adults who favor simple, low-complexity exercises [34]. Adherence serves as a crucial determinant in the effectiveness of home-based interventions. Garcia-Roca et al. performed a systematic review indicating that adherence rates to home exercise programs exhibit significant variability, generally increasing when exercises are simple to execute and necessitate minimal oversight [35]. Kumar observed that patients expressed greater satisfaction and perceived ease of execution with FE relative to alternative balance training methods, likely fostering sustained engagement and improved outcomes [36].

Eckert et al. conducted a cost-effectiveness analysis demonstrating that home-based balance interventions, such as FE, effectively reduce healthcare expenditures by decreasing the incidence of falls and the related treatment costs [37]. In addition, Nguyen et al. conducted a meta-analysis of fall prevention programs, determining that interventions focused on accessibility and patient adherence, such as FE, produce the most significant economic benefits by averting expensive fall-related injuries [38]. The findings highlight the necessity of accounting for confounding variables, including participant adherence and the simplicity of exercise execution, when assessing intervention outcomes. FE's straightforward and domestic characteristics likely improve adherence; however, they may also lead to inconsistencies in exercise quality, indicating a necessity for strategies that promote proper practice and sustained motivation.

While several studies have reported comparable improvements in motor function through FE [28], the variations in methods and sample populations across studies complicate direct comparisons. Variations in FE's frequency, duration, and delivery method may account for some observed outcome discrepancies across studies [39–41]. An in-depth examination of the intervention durations within the selected studies uncovers significant implications for their effects on outcomes. Interventions generally lasted 5–6 weeks, with an average of three weekly sessions. The observed variation in effect sizes across studies may be partially attributed to differences in the total exposure time and session duration, despite the standardization provided by this consistency. For example, research conducted by Tabatabai Asl et al., which utilized a 6-week duration with 60-min sessions, demonstrated significantly greater enhancements in balance and a reduction in fall risk compared to shorter or less intensive interventions. This indicates a potential dose–response relationship, where the increased duration or frequency of interventions may enhance neuromuscular adaptation, proprioceptive feedback, and confidence in mobility. The lack of long-term interventions, particularly those surpassing 8 weeks, constrains our comprehension of whether the advantages of FEs stabilize or enhance over time. Research in related fields, including Tai Chi and Otago programs, indicates that prolonged engagement (8–12 weeks) results in lasting enhancements in balance and fall prevention [20]. Integrating extended FE protocols in the forthcoming research could elucidate whether a comparable trend is observed in this context. Consequently, in addition to frequency and type, duration must be considered a crucial design variable when assessing or recommending FE interventions for elderly individuals with neurological disorders [31, 42].

Nevertheless, the general trend supports the effectiveness of FE, particularly for enhancing balance in individuals with Parkinson's disease and other neurological disorders, a finding consistent with the broader literature on physical interventions for balance improvement [29]. An RCT conducted with institutionalized elderly adults demonstrated that both functional exercises and aerobic walking significantly enhanced static and dynamic balance, revealing no notable differences between the two groups [14]. Similarly, Solak et al. illustrated that a 4-week home-based FE program for postmenopausal women experiencing balance impairments significantly enhanced functional reach, single-leg stance, timed up and go, and overall quality of life. The findings align with the current review and reinforce that structured coordination exercises may be practical in geriatric rehabilitation strategies [43].

A salient finding from the present review was the observed enhancement in social participation and mental well-being among subjects undergoing FE, even though FE primarily focuses on physical outcomes. Some studies, including those by Vafaeenasab et al. and Tep et al., noted secondary benefits, including reduced fear of falling and increased confidence in mobility. These findings are noteworthy because they suggest that FE may offer psychological benefits beyond the physical improvements traditionally associated with exercise interventions, thereby highlighting the potential of FE to contribute to a holistic approach to the care of elderly individuals with neurological disorders [14, 27].

Regarding motor function, FE has been demonstrated to enhance muscle strength and motor coordination [14, 28]. For instance, in an investigation of older women, Moradi et al. reported that significant improvements in functional mobility and motor coordination were observed in the group following participation in a 6-week FE program [28]. The efficacy of FE is contingent upon the specific underlying neurological disorder. In individuals diagnosed with MS, Afrasiabifar et al. demonstrated that while both Cawthorne–Cooksey and FEs enhanced balance, the former resulted in a more pronounced improvement in the Berg Balance Scale. This indicates vestibular-targeted interventions may be more effective than FEs in populations experiencing sensory integration dysfunction. The observed differences highlight the necessity of customizing exercise interventions to align with the unique neurological deficits of patients [23].

An additional critical element is the psychological advantage linked to balance training. While the primary focus is physical function, numerous studies indicate that FE interventions enhance confidence and decrease the fear of falling [43, 44]. The secondary outcomes could improve physical activity and social engagement, contributing to overall well-being. However, the repetitive characteristics of FE could pose challenges to motivation. In response to this issue, contemporary methodologies have integrated virtual reality and interactive feedback systems. Kaminska et al. demonstrated that virtual reality-based balance training markedly decreased the risk of falls while increasing engagement and enjoyment in older adults [45].

These unexpected findings could have important implications for future interventions. They suggest that it may be beneficial to explore the integration of FE with cognitive–behavioral techniques or other psychosocial interventions to maximize the physical and psychological outcomes for elderly individuals at risk of falls.

## 5. Study Limitations

It is essential to recognize the limitations of this review when interpreting the findings. First, the methodological quality of several studies was moderate, with some lacking control groups or randomization. This diminishes the strength of the evidence and warrants caution in concluding. Additionally, many studies featured small sample sizes, which restricts the generalizability of the results and increases the risk of random outcomes and type I and II errors. Furthermore, the absence of long-term follow-up data in several studies hinders the evaluation of the sustainability of the benefits of FE over time. The variation in intervention types, such as group versus individual FE and home-based versus supervised exercise, complicates the identification of optimal delivery conditions for FE. Future research should prioritize the standardization of intervention protocols and include long-term follow-up to assess the durability of the effects. The initial search in PubMed was filtered to include only “Free full-text” articles. Although this may restrict access to specific subscription-based articles within this database, the limitation was addressed by exploring additional prominent databases free from such constraints, utilizing a snowballing method to uncover further research through reference lists, and reaching out to authors for full texts that were not readily available.

## 6. Conclusion

This systematic review endorses the utilization of FE as an effective intervention for improving dynamic balance and motor function in elderly individuals with neurological disorders. The accessibility, cost-effectiveness, and adaptability of FE position it as a promising option for a wide range of older adults, particularly those at risk of falls. While further research is necessary to substantiate these findings and explore the psychological benefits of FE, current evidence suggests that this straightforward yet effective intervention could significantly contribute to fall prevention and enhance the overall quality of life for elderly individuals with neurological conditions.

Despite the limitations of this review, its findings highlight the need for continued research in this domain. Specifically, studies involving more extensive and diverse populations, employing rigorous designs such as RCTs, are essential to confirm the efficacy of FE in enhancing dynamic balance and motor function. Additionally, future research should evaluate the impact of FE on mental health outcomes, particularly in reducing fear of falling and increasing social participation. Finally, investigating the combination of FE with other interventions, such as cognitive–behavioral therapy or social support programs, could offer a more comprehensive approach to fall prevention and rehabilitation for elderly individuals with neurological conditions.

## Figures and Tables

**Table 1:** Combination of search terms used with operators and databases.

Database	English search term combination
Google scholar	(Frenkel exercises) AND (dynamic balance) AND (motor function OR gait OR postural balance) AND (elderly OR aged) AND (neurological disorders OR disorders, neurological OR disorder, neurological OR disease, nervous system OR diseases, nervous system OR nervous system disease OR nervous system disorders OR disorder, nervous system OR disorders, nervous system OR nervous system disorder OR neurologic disorders OR disorder, neurologic OR disorders, neurologic OR neurologic disorder)

PubMed	(((((((((((((((((Frenkel exercises [title/abstract]) AND (dynamic balance [title/abstract])) AND (motor function [title/abstract])) OR (gait [title/abstract])) OR (postural balance [title/abstract])) AND (elderly [title/abstract])) OR (aged [title/abstract])) AND (neurological disorders [title/abstract])) OR (disorders, neurological [title/abstract])) OR (disorder, neurological [title/abstract])) OR (disease, nervous system [title/abstract])) OR (diseases, nervous system [title/abstract])) OR (nervous system disease [title/abstract])) OR (nervous system disorders [title/abstract])) OR (disorder, nervous system [title/abstract])) OR (disorders, nervous system [title/abstract])) OR (nervous system disorder [title/abstract])) OR (neurologic disorders [title/abstract])) OR (disorder, neurologic [title/abstract])) OR (disorders, neurologic [title/abstract])) OR (neurologic disorder [title/abstract]) Filters: Last 5 years, free full text, observational study, randomized controlled trial, humans

Scopus	(TITLE-ABS-KEY (Frenkel exercises) AND TITLE-ABS-KEY (dynamic balance) AND TITLE-ABS-KEY (motor function OR gait OR postural balance) AND TITLE-ABS-KEY (elderly OR aged) AND TITLE-ABS-KEY (neurological disorders OR disorders, neurological OR disorder, neurological OR disease, nervous system OR diseases, nervous system OR nervous system disease OR nervous system disorders OR disorder, nervous system OR disorders, nervous system OR nervous system disorder OR neurologic disorders OR disorder, neurologic OR disorders, neurologic OR neurologic disorder)) AND (LIMIT-TO (PUBYEAR, 2020–2025) AND LIMIT-TO (DOCTYPE, “ar”) AND LIMIT-TO (SUBJAREA, “MEDI”))

Web of Science	TS = (“Frenkel exercises”) AND TS = (“dynamic balance”) AND TS = (“motor function” OR “gait” OR “postural balance”) AND TS = (“elderly” OR “aged”) AND TS = (“neurological disorders” OR “disorders, neurological” OR “disorder, neurological” OR “disease, nervous system” OR “diseases, nervous system” OR “nervous system disease” OR “nervous system disorders” OR “disorder, nervous system” OR “disorders, nervous system” OR “nervous system disorder” OR “neurologic disorders” OR “disorder, neurologic” OR “disorders, neurologic” OR “neurologic disorder”) AND PY = (2020–2025) AND DT = (Article) AND SU = (Medicine)

ScienceDirect	“Frenkel exercises” AND “dynamic balance” AND (“motor function” OR “gait” OR “postural balance”) AND (“elderly” OR “aged”) AND (“neurological disorders” OR “disorders, neurological” OR “disorder, neurological” OR “disease, nervous system” OR “diseases, nervous system” OR “nervous system disease” OR “nervous system disorders” OR “disorder, nervous system” OR “disorders, nervous system” OR “nervous system disorder”) AND PUBYEAR > 2019 AND DOCTYPE (ar) AND SUBJAREA(MEDI)

Cochrane Library	#1. Frenkel exercises
	#2. Dynamic balance
	#3. Motor function
	#4. Gait
	#5. Postural balance
	#6. #1 OR #2 OR #3 OR #4 OR #5
	#7. Elderly
	#8. Aged
	#9. #7 OR #8
	#10. Neurological disorders
	#11. Disorders, neurological OR disorder, neurological
	#12. Disease, nervous system OR diseases, nervous system
	#13. Nervous system disease OR nervous system disorders
	#14. Disorder, nervous system OR disorders, nervous system
	#15. Nervous system disorder
	#16. Neurologic disorders OR disorder, neurologic
	#17. Disorders, neurologic OR neurologic disorder
	#18. #10 OR #11 OR #12 OR #13 OR #14 OR #15 OR #16 OR #17
	#19. #6 AND #9 AND #18
	Filters: Published in last 5 years, trials, reviews

*Note:* The table presents a combination of search terms and Boolean operators used to identify relevant studies in the systematic review. It also shows the databases searched and the inclusion criteria applied to select studies for the review.

**Table 2:** PICOS framework: Frenkel exercises in the elderly.

P (population)	I (intervention)	C (comparison)	O (outcome)	S (study design)
Elderly individuals (aged ≥ 65 years) diagnosed with neurological disorders (e.g., Parkinson's disease, multiple sclerosis, stroke, Huntington's disease, myasthenia gravis, muscular dystrophy, Guillain–Barré syndrome, and amyotrophic lateral sclerosis)	Frenkel exercises, consisting of repetitive, coordinated movements to enhance proprioception, balance, and motor coordination	Elderly individuals with similar conditions receiving no intervention or alternative exercise interventions (e.g., Tai Chi, Otago exercise program, or virtual reality-based training)	Primary outcomes: Dynamic balance (e.g., Berg Balance Scale, timed up, and go test), motor function (e.g., Unified Parkinson's Disease Rating Scale, muscle strength tests); secondary outcome: Fall risk reduction (e.g., number of falls, falls efficacy scale)	RCTs, cohort studies, case–control studies, and cross-sectional studies

*Note:* The table illustrates the application of the PICOS framework to evaluate the effectiveness of Frenkel exercises in elderly individuals with neurological disorders. The table outlines the population (elderly individuals with neurological disorders), intervention (Frenkel exercises), comparison (control groups), outcome (improvement in dynamic balance and motor function), and study design (randomized controlled trials, cohort studies, case–control studies, and cross-sectional studies).

**Table 3:** Characteristics of studies included in the systematic review.

Author (year)	Study design	Population	Intervention (Frenkel)	Control group	Outcome measures	Key findings
1. Tep et al. (2023) [27]	Comparative study	30 elderly adults (15 in each group)	Frenkel exercises in supine, sitting, and standing positions	Home-based Otago exercise program	Timed up and go (TUG), fall efficacy scale (FES)	Both interventions improved balance. Otago was more effective in reducing the fear of falls.
2. Vafaeenasab et al. (2020) [14]	Randomized controlled study	48 elderly residents of nursing homes	Frenkel exercises, three sessions/week for 5 weeks	Aerobic exercises (walking)	Sharpened Romberg, get up and go	Both groups showed improvement in static and dynamic balance, with no significant difference between them.
3. Moradi et al. (2021) [28]	Quasi-experimental pre–post-study	30 older women with kyphosis	Frenkel exercises, three sessions/week for 5 weeks	No intervention	Kinematic parameters, 180° rotation test, Berg Balance Scale (BBS)	Significant improvement in balance, posture stability, and gait in the intervention group.
4. Ray et al. (2023) [29]	Comparative study	32 elderly (16 per group) with medium fall risk	Frenkel exercises, three sessions/week for 5 weeks	Cawthorne-Cooksey exercises	BBS	Both interventions equally improved balance, with no significant difference between groups.
5. Dash et al. (2024) [30]	Randomized controlled study	30 elderly adults (15 per group) with a history of falls	Frenkel exercises in supine, sitting, and standing positions	Home-based exercises (strengthening and balance)	TUG, FES	Both interventions improved balance. Home-based exercises were more effective in reducing the fear of falls.
6. Tabatabai Asl et al. (2022) [31]	Quasi-experimental study	28 older men and women with a history of falls in nursing homes	Combination of Cawthorne–Cooksey and Frenkel exercises, 6 weeks, three sessions/week, 60 min per session	No intervention	BBS, TUG	Significant improvement in functional balance and fall risk in the intervention group.

*Note:* The table summarizes the key characteristics of the studies included in the systematic review, including study design, sample size, participant characteristics (age, gender, and type of neurological disorder), duration of intervention, and outcome measures used. It provides an overview of each study's methodological quality and main findings.

**Table 4:** Assessment of selected studies using the MMAT.

Study	Research questions defined	Data adequately address research question	Study design appropriate	Participants representative	Outcome measures appropriate	Outcome data complete	Confounding variables controlled	Adherence ensured	Risk of bias	%
Tep et al. (2023)	✔	✔	✔	✔	✔	✔	✔	✔	✔	100 (9/9)
Ray et al. (2023)	✔	✔	✔	×	✔	✔	✔	✔	✔	88 (8/9)
Tabatabai Asl et al. (2022)	✔	×	✔	✔	✔	✔	✔	✔	✔	88 (8/9)
Moradi et al. (2021)	✔	✔	✔	✔	✔	✔	✔	×	✔	88 (8/9)
Vafaenasab et al. (2020)	✔	✔	✔	✔	✔	✔	✔	✔	✔	100 (9/9)
Dash et al. (2024)	✔	✔	✔	✔	×	✔	✔	✔	✔	88 (8/9)

*Note:* The table presents the assessment of the selected studies using the Mixed Methods Appraisal Tool (MMAT). The table evaluates the methodological quality of each study based on five criteria: study design, sampling method, outcome measures, data collection procedures, and analysis techniques. Studies are classified as high quality if they meet at least 50% of the criteria, based on the MMAT evaluation.

## Data Availability

Data sharing is not applicable to this article as no new data were created or analyzed in this study.

## Nomenclature

FEs: Frenkel Exercises
PRISMA: Preferred Reporting Items for Systematic Reviews and Meta-Analyses
MeSH: Medical Subject Headings
PICOS: Population, Intervention, Comparison, Outcome, Study Design
MMAT: Mixed Methods Appraisal Tool
RCTs: Randomized Controlled Trials
BBS: Berg Balance Scale
TUG: Timed Up and Go Test
FES: Fall Efficacy Scale
UPDRS: Unified Parkinson's Disease Rating Scale
WHO: World Health Organization
OEP: Otago Exercise Program
MS: Multiple Sclerosis
AI: Artificial Intelligence
